# [4-Chloro-*N*′-(3-meth­oxy-2-oxidobenzyl­idene)benzohydrazidato]dimethyl­tin(IV)

**DOI:** 10.1107/S1600536808040786

**Published:** 2008-12-10

**Authors:** Jichun Cui, Yanling Qiao, Handong Yin

**Affiliations:** a College of Chemistry and Chemical Engineering, Liaocheng University, Shandong 252059, People’s Republic of China

## Abstract

In the title mol­ecule, [Sn(CH_3_)_2_(C_15_H_11_ClN_2_O_3_)], the two benzene rings form a dihedral angle of 6.37 (2)°. The Sn atom is coordinated by one N [Sn—N = 2.187 (3) Å], two O [Sn—O = 2.123 (3) and 2.174 (3) Å] and two C [Sn—C = 2.096 (4) and 2.101 (4) Å] atoms in a distorted trigonal-bipyramidal geometry. The crystal packing exhibits weak inter­molecular C—H⋯O hydrogen bonds, which link the mol­ecules into centrosymmetric dimers with an Sn⋯Sn separation of 4.330 (6) Å, and π–π inter­actions [centroid–centroid distance of 3.690 (5) Å between the benzene rings of neighbouring mol­ecules].

## Related literature

For a related crystal structure, see Hong *et al.* (2006 ▶).
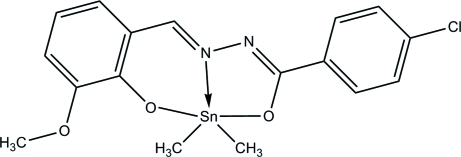


## Experimental

### 

#### Crystal data

[Sn(CH_3_)_2_(C_15_H_11_ClN_2_O_3_)]
*M* *_r_* = 451.47
Monoclinic, 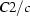

*a* = 30.015 (3) Å
*b* = 9.5039 (10) Å
*c* = 13.5615 (18) Å
β = 113.189 (2)°
*V* = 3556.0 (7) Å^3^
*Z* = 8
Mo *K*α radiation
μ = 1.60 mm^−1^
*T* = 298 (2) K
0.50 × 0.20 × 0.08 mm

#### Data collection

Siemens SMART CCD area-detector diffractometer
Absorption correction: multi-scan (*SADABS*; Sheldrick, 1996 ▶) *T*_min_ = 0.501, *T*_max_ = 0.882
8568 measured reflections
3113 independent reflections
2392 reflections with *I* > 2σ(*I*)
*R*_int_ = 0.061

#### Refinement

*R*[*F* ^2^ > 2σ(*F* ^2^)] = 0.029
*wR*(*F* ^2^) = 0.080
*S* = 1.00
3113 reflections
220 parameters
H-atom parameters constrained
Δρ_max_ = 0.66 e Å^−3^
Δρ_min_ = −0.57 e Å^−3^

### 

Data collection: *SMART* (Siemens, 1996 ▶); cell refinement: *SAINT* (Siemens, 1996 ▶); data reduction: *SAINT*; program(s) used to solve structure: *SHELXS97* (Sheldrick, 2008 ▶); program(s) used to refine structure: *SHELXL97* (Sheldrick, 2008 ▶); molecular graphics: *SHELXTL* (Sheldrick, 2008 ▶); software used to prepare material for publication: *SHELXTL*.

## Supplementary Material

Crystal structure: contains datablocks I, global. DOI: 10.1107/S1600536808040786/cv2482sup1.cif

Structure factors: contains datablocks I. DOI: 10.1107/S1600536808040786/cv2482Isup2.hkl

Additional supplementary materials:  crystallographic information; 3D view; checkCIF report

## Figures and Tables

**Table 1:** Hydrogen-bond geometry (Å, °)

*D*—H⋯*A*	*D*—H	H⋯*A*	*D*⋯*A*	*D*—H⋯*A*
C15—H15*B*⋯O1^i^	0.96	2.53	3.290 (6)	136

Symmetry code: (i) 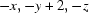.

**Fig. 1.**
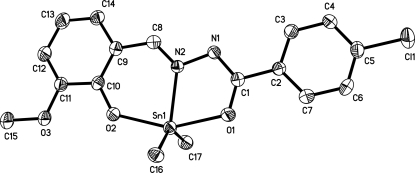
The molecular structure of (I), showing 30% probability displacement ellipsoids and the atom-numbering scheme. H atoms have been omitted for clarity.

## supplementary crystallographic information

### Comment

The molecular structure of the title compound, (I), is shown in Fig. 1. The Sn atom has distorted trigonal-bipyramidal coordination geometry, with atoms O1 and O2 in axial positions [O1—Sn1—O2 = 153.92 (11)°] and the atoms C16, C17 and N2 in equatorial positions. The sum of the equatorial angles C16—Sn1—C17, C16—Sn1—N2 and C17—Sn—N2 is 359.4 (1) °, indicating approximate coplanarity for these atoms. The Sn1—N2 bond length is 2.187 (3) Å close to the sum of the non-polar covalent radii 2.15 Å, indicating a strong Sn—N interaction. The Sn-O coordinating bond lengths are close to those in the reported compound [Sn(C_6_H_5_)_2_(C_14_H_10_N_2_O_3_)].C_2_H_6_O (Hong *et al.*, 2006). The crystal packing exhibits weak intermolecular C—H···O hydrogen bonds (Table 2), which link the molecules into centrosymmetric dimers with Sn···Sn separation of 4.330 (6) Å, and π–π interactions proved by short distance of 3.690 (5) between the centroids of benzene rings from the neighbouring molecules (Table 1).

### Experimental

The reaction was carried out under nitrogen atmosphere. *o*-vanillin 4-chlorobenzhydrazone(1 mmol) and sodium ethoxide (1.2 mmol) were added to the solution of benzene(30 ml) in a Schlenk flask and stirred for 0.5 h. Dimethyltin dichloride (1 mmol) was then added to the reactor and the reaction mixture was stirred for 4 h at 313 K.The resulting clear solution was evaporated under vacuum. The product was crystallized from a mixture of dichloromethane/methanol (1:1). Analysis calculated (75%) for C_17_H_17_ClN_2_O_3_Sn (Mr = 451.47): C, 45.22; H, 3.80; N, 6.20, found: C, 45.09; H, 3.76; N, 6.35.

### Refinement

All H atoms attached to C atoms were fixed geometrically and treated as riding with, with aromatic C—H distances of 0.93 Å, methyl C—H distances of 0.96 Å. The *U*_iso_(H) values were set at 1.5*U*_iso_(C) for the methyl H atoms, and at 1.2*U*_iso_(C) for the other H atoms.

### Crystal data

**Table:**

[Sn(CH_3_)_2_(C_15_H_11_ClN_2_O_3_)]	*F*(000) = 1792
*M**_r_* = 451.47	*D*_x_ = 1.687 Mg m^−^^3^
Monoclinic, *C*2/*c*	Mo *K*α radiation, λ = 0.71073 Å
*a* = 30.015 (3) Å	Cell parameters from 4052 reflections
*b* = 9.5039 (10) Å	θ = 2.3–28.1°
*c* = 13.5615 (18) Å	µ = 1.60 mm^−^^1^
β = 113.189 (2)°	*T* = 298 K
*V* = 3556.0 (7) Å^3^	Block, orange
*Z* = 8	0.50 × 0.20 × 0.08 mm

### Data collection

**Table:**

Siemens SMART CCD area-detector diffractometer	3113 independent reflections
Radiation source: fine-focus sealed tube	2392 reflections with *I* > 2σ(*I*)
graphite	*R*_int_ = 0.061
φ and ω scans	θ_max_ = 25.0°, θ_min_ = 2.3°
Absorption correction: multi-scan (*SADABS*; Sheldrick, 1996)	*h* = −35→35
*T*_min_ = 0.501, *T*_max_ = 0.882	*k* = −10→11
8568 measured reflections	*l* = −8→16

### Refinement

**Table:**

Refinement on *F*^2^	Primary atom site location: structure-invariant direct methods
Least-squares matrix: full	Secondary atom site location: difference Fourier map
*R*[*F*^2^ > 2σ(*F*^2^)] = 0.029	Hydrogen site location: inferred from neighbouring sites
*wR*(*F*^2^) = 0.080	H-atom parameters constrained
*S* = 1.00	*w* = 1/[σ^2^(*F*_o_^2^) + (0.0351*P*)^2^ + 0.1164*P*] where *P* = (*F*_o_^2^ + 2*F*_c_^2^)/3
3113 reflections	(Δ/σ)_max_ = 0.001
220 parameters	Δρ_max_ = 0.66 e Å^−^^3^
0 restraints	Δρ_min_ = −0.56 e Å^−^^3^

### Fractional atomic coordinates and isotropic or equivalent isotropic displacement parameters (Å^2^)

**Table:**

	*x*	*y*	*z*	*U*_iso_*/*U*_eq_	
Sn1	0.032667 (8)	0.79772 (3)	0.059696 (18)	0.03884 (12)	
Cl1	0.23989 (4)	0.12286 (15)	0.38025 (11)	0.0824 (4)	
N1	0.03655 (10)	0.5011 (3)	0.1544 (2)	0.0386 (7)	
N2	0.00348 (9)	0.6097 (3)	0.1058 (2)	0.0343 (7)	
O1	0.08954 (9)	0.6394 (3)	0.1128 (2)	0.0516 (7)	
O2	−0.04042 (8)	0.8667 (3)	0.0035 (2)	0.0481 (7)	
O3	−0.11949 (8)	1.0208 (3)	−0.0695 (2)	0.0593 (8)	
C1	0.07925 (12)	0.5289 (4)	0.1536 (3)	0.0375 (9)	
C2	0.11826 (12)	0.4249 (4)	0.2063 (3)	0.0373 (9)	
C3	0.11002 (14)	0.3030 (4)	0.2523 (3)	0.0452 (10)	
H3	0.0788	0.2837	0.2472	0.054*	
C4	0.14663 (14)	0.2095 (4)	0.3054 (3)	0.0484 (10)	
H4	0.1405	0.1282	0.3361	0.058*	
C5	0.19244 (14)	0.2392 (5)	0.3119 (3)	0.0515 (11)	
C6	0.20167 (14)	0.3569 (5)	0.2656 (4)	0.0706 (14)	
H6	0.2328	0.3747	0.2696	0.085*	
C7	0.16426 (14)	0.4496 (5)	0.2123 (4)	0.0603 (12)	
H7	0.1704	0.5297	0.1803	0.072*	
C8	−0.03937 (12)	0.5902 (4)	0.1040 (3)	0.0394 (9)	
H8	−0.0445	0.5060	0.1330	0.047*	
C9	−0.07999 (13)	0.6841 (4)	0.0622 (3)	0.0392 (9)	
C10	−0.07884 (13)	0.8150 (4)	0.0152 (3)	0.0396 (9)	
C11	−0.12244 (12)	0.8960 (4)	−0.0225 (3)	0.0446 (10)	
C12	−0.16337 (14)	0.8486 (5)	−0.0114 (3)	0.0570 (12)	
H12	−0.1911	0.9040	−0.0351	0.068*	
C13	−0.16372 (15)	0.7186 (5)	0.0348 (4)	0.0655 (14)	
H13	−0.1917	0.6872	0.0417	0.079*	
C14	−0.12297 (13)	0.6366 (5)	0.0703 (3)	0.0529 (11)	
H14	−0.1236	0.5488	0.1001	0.063*	
C15	−0.16273 (14)	1.1007 (5)	−0.1172 (4)	0.0785 (16)	
H15A	−0.1874	1.0434	−0.1683	0.118*	
H15B	−0.1566	1.1810	−0.1529	0.118*	
H15C	−0.1733	1.1315	−0.0626	0.118*	
C16	0.03486 (14)	0.8041 (4)	−0.0928 (3)	0.0450 (10)	
H16A	0.0310	0.8996	−0.1180	0.068*	
H16B	0.0091	0.7475	−0.1416	0.068*	
H16C	0.0654	0.7683	−0.0887	0.068*	
C17	0.05918 (14)	0.9110 (4)	0.2044 (3)	0.0508 (11)	
H17A	0.0896	0.8717	0.2515	0.076*	
H17B	0.0363	0.9053	0.2378	0.076*	
H17C	0.0637	1.0077	0.1901	0.076*	

### Atomic displacement parameters (Å^2^)

**Table:**

	*U*^11^	*U*^22^	*U*^33^	*U*^12^	*U*^13^	*U*^23^
Sn1	0.03741 (16)	0.0408 (2)	0.04022 (17)	0.00259 (12)	0.01728 (12)	0.00166 (12)
Cl1	0.0532 (6)	0.0705 (9)	0.1054 (10)	0.0202 (6)	0.0120 (7)	0.0138 (8)
N1	0.0381 (16)	0.0376 (19)	0.0420 (17)	0.0032 (14)	0.0179 (13)	0.0047 (15)
N2	0.0349 (15)	0.0350 (18)	0.0347 (15)	−0.0004 (14)	0.0156 (13)	−0.0008 (14)
O1	0.0453 (15)	0.0475 (18)	0.0701 (18)	0.0051 (13)	0.0315 (14)	0.0148 (16)
O2	0.0381 (13)	0.0485 (18)	0.0603 (16)	0.0043 (12)	0.0222 (13)	0.0120 (14)
O3	0.0347 (14)	0.0504 (19)	0.086 (2)	0.0068 (13)	0.0167 (14)	0.0133 (17)
C1	0.041 (2)	0.040 (2)	0.0346 (19)	0.0022 (17)	0.0176 (16)	−0.0034 (17)
C2	0.0383 (19)	0.036 (2)	0.040 (2)	−0.0003 (16)	0.0178 (16)	−0.0031 (17)
C3	0.042 (2)	0.045 (3)	0.048 (2)	−0.0001 (19)	0.0167 (18)	−0.002 (2)
C4	0.048 (2)	0.041 (3)	0.054 (2)	0.0014 (19)	0.0167 (19)	0.008 (2)
C5	0.043 (2)	0.048 (3)	0.055 (2)	0.008 (2)	0.0104 (19)	−0.002 (2)
C6	0.033 (2)	0.073 (3)	0.101 (4)	0.002 (2)	0.022 (2)	0.019 (3)
C7	0.047 (2)	0.055 (3)	0.082 (3)	0.001 (2)	0.029 (2)	0.018 (3)
C8	0.045 (2)	0.041 (2)	0.0353 (19)	−0.0024 (18)	0.0191 (16)	−0.0051 (17)
C9	0.0394 (19)	0.048 (3)	0.0317 (18)	0.0020 (18)	0.0152 (16)	−0.0009 (17)
C10	0.0358 (19)	0.047 (3)	0.0346 (19)	−0.0015 (17)	0.0128 (16)	−0.0051 (18)
C11	0.039 (2)	0.045 (3)	0.047 (2)	0.0023 (19)	0.0141 (17)	−0.001 (2)
C12	0.038 (2)	0.063 (3)	0.071 (3)	0.006 (2)	0.022 (2)	0.011 (3)
C13	0.039 (2)	0.087 (4)	0.076 (3)	0.002 (2)	0.029 (2)	0.019 (3)
C14	0.042 (2)	0.064 (3)	0.056 (2)	−0.001 (2)	0.0234 (19)	0.011 (2)
C15	0.041 (2)	0.067 (3)	0.113 (4)	0.011 (2)	0.014 (3)	0.023 (3)
C16	0.053 (2)	0.042 (2)	0.045 (2)	0.0016 (19)	0.0245 (18)	0.0022 (19)
C17	0.056 (2)	0.047 (3)	0.044 (2)	−0.005 (2)	0.0149 (19)	−0.002 (2)

### Geometric parameters (Å, °)

**Table:**

Sn1—C16	2.096 (4)	C6—H6	0.9300
Sn1—C17	2.101 (4)	C7—H7	0.9300
Sn1—O2	2.123 (2)	C8—C9	1.436 (5)
Sn1—O1	2.174 (3)	C8—H8	0.9300
Sn1—N2	2.187 (3)	C9—C10	1.404 (5)
Cl1—C5	1.752 (4)	C9—C14	1.412 (5)
N1—C1	1.313 (4)	C10—C11	1.428 (5)
N1—N2	1.403 (4)	C11—C12	1.371 (5)
N2—C8	1.290 (4)	C12—C13	1.387 (6)
O1—C1	1.281 (4)	C12—H12	0.9300
O2—C10	1.319 (4)	C13—C14	1.368 (6)
O3—C11	1.365 (5)	C13—H13	0.9300
O3—C15	1.421 (5)	C14—H14	0.9300
C1—C2	1.483 (5)	C15—H15A	0.9600
C2—C7	1.371 (5)	C15—H15B	0.9600
C2—C3	1.383 (5)	C15—H15C	0.9600
C3—C4	1.376 (5)	C16—H16A	0.9600
C3—H3	0.9300	C16—H16B	0.9600
C4—C5	1.372 (6)	C16—H16C	0.9600
C4—H4	0.9300	C17—H17A	0.9600
C5—C6	1.364 (6)	C17—H17B	0.9600
C6—C7	1.386 (6)	C17—H17C	0.9600
			
Sn1···Sn1^i^	4.3302 (6)	Cg1···Cg2^ii^	3.690 (5)
			
C16—Sn1—C17	139.41 (15)	C14—C13—H13	119.9
C16—Sn1—O2	93.81 (13)	C12—C13—H13	119.9
C17—Sn1—O2	97.74 (13)	C13—C14—C9	120.5 (4)
C16—Sn1—O1	91.21 (13)	C13—C14—H14	119.7
C17—Sn1—O1	94.93 (13)	C9—C14—H14	119.7
O2—Sn1—O1	153.92 (11)	O3—C15—H15A	109.5
C16—Sn1—N2	118.54 (12)	O3—C15—H15B	109.5
C17—Sn1—N2	101.45 (14)	H15A—C15—H15B	109.5
O2—Sn1—N2	83.18 (10)	O3—C15—H15C	109.5
O1—Sn1—N2	71.99 (10)	H15A—C15—H15C	109.5
C16—Sn1—Sn1^i^	77.92 (10)	H15B—C15—H15C	109.5
C17—Sn1—Sn1^i^	81.00 (11)	Sn1—C16—H16A	109.5
O2—Sn1—Sn1^i^	48.68 (7)	Sn1—C16—H16B	109.5
O1—Sn1—Sn1^i^	156.79 (7)	H16A—C16—H16B	109.5
N2—Sn1—Sn1^i^	131.22 (7)	Sn1—C16—H16C	109.5
C1—N1—N2	111.2 (3)	H16A—C16—H16C	109.5
C8—N2—N1	114.8 (3)	H16B—C16—H16C	109.5
C8—N2—Sn1	128.4 (3)	Sn1—C17—H17A	109.5
N1—N2—Sn1	116.5 (2)	Sn1—C17—H17B	109.5
C1—O1—Sn1	114.8 (2)	H17A—C17—H17B	109.5
C10—O2—Sn1	132.1 (2)	Sn1—C17—H17C	109.5
C11—O3—C15	117.4 (3)	H17A—C17—H17C	109.5
O1—C1—N1	124.8 (3)	H17B—C17—H17C	109.5
O1—C1—C2	118.4 (3)	C3—CG1—C5	119.9 (3)
N1—C1—C2	116.7 (3)	C3—CG1—C7	119.8 (2)
C7—C2—C3	118.1 (4)	C5—CG1—C7	120.3 (2)
C7—C2—C1	120.0 (4)	C3—CG1—C6	179.4 (3)
C3—C2—C1	121.9 (3)	C5—CG1—C6	59.6 (3)
C2—C3—C4	121.9 (4)	C7—CG1—C6	60.6 (2)
C2—C3—H3	119.0	C3—CG1—C4	60.1 (2)
C4—C3—H3	119.0	C5—CG1—C4	59.8 (2)
C5—C4—C3	118.3 (4)	C7—CG1—C4	179.6 (3)
C5—C4—H4	120.8	C6—CG1—C4	119.4 (3)
C3—C4—H4	120.8	C3—CG1—C2	60.4 (2)
C6—C5—C4	121.3 (4)	C5—CG1—C2	179.2 (2)
C6—C5—Cl1	119.4 (3)	C7—CG1—C2	59.5 (2)
C4—C5—Cl1	119.3 (4)	C6—CG1—C2	120.1 (3)
C5—C6—C7	119.5 (4)	C4—CG1—C2	120.5 (2)
C5—C6—H6	120.3	C3—CG1—CG2^ii^	91.56 (16)
C7—C6—H6	120.3	C5—CG1—CG2^ii^	102.81 (18)
C2—C7—C6	120.8 (4)	C7—CG1—CG2^ii^	75.46 (19)
C2—C7—H7	119.6	C6—CG1—CG2^ii^	88.2 (2)
C6—C7—H7	119.6	C4—CG1—CG2^ii^	104.11 (17)
N2—C8—C9	127.1 (4)	C2—CG1—CG2^ii^	77.95 (14)
N2—C8—H8	116.4	C11—CG2—C12	59.4 (2)
C9—C8—H8	116.4	C11—CG2—C14	178.1 (2)
C10—C9—C14	120.4 (3)	C12—CG2—C14	118.9 (2)
C10—C9—C8	124.2 (4)	C11—CG2—C13	119.3 (2)
C14—C9—C8	115.5 (4)	C12—CG2—C13	59.9 (2)
O2—C10—C9	124.3 (3)	C14—CG2—C13	59.0 (2)
O2—C10—C11	118.5 (4)	C11—CG2—C9	120.8 (2)
C9—C10—C11	117.2 (3)	C12—CG2—C9	179.5 (2)
O3—C11—C12	124.2 (3)	C14—CG2—C9	61.0 (2)
O3—C11—C10	114.6 (3)	C13—CG2—C9	120.0 (2)
C12—C11—C10	121.2 (4)	C11—CG2—C10	61.1 (2)
C11—C12—C13	120.6 (4)	C12—CG2—C10	120.4 (2)
C11—C12—H12	119.7	C14—CG2—C10	120.6 (2)
C13—C12—H12	119.7	C13—CG2—C10	179.6 (3)
C14—C13—C12	120.1 (4)	C9—CG2—C10	59.7 (2)

Symmetry codes: (i) −*x*, −*y*+2, −*z*; (ii) −*x*, −*y*+1, −*z*.

### Hydrogen-bond geometry (Å, °)

**Table:**

*D*—H···*A*	*D*—H	H···*A*	*D*···*A*	*D*—H···*A*
C15—H15B···O1^i^	0.96	2.53	3.290 (6)	136

Symmetry codes: (i) −*x*, −*y*+2, −*z*.
